# The Hospital

**Published:** 1917-10-20

**Authors:** 


### INSTITUTIONAL WORKER

**Being a Special Supplement to "The Hospital"**

### OUR BUREAU OF INFORMATION.

### Rules for Correspondents.

**1. Every letter must be accompanied by the coupon to be cut from the back cover (inside page) of THE HOSPITAL, current issue, and must contain the name and address of the correspondent with pseudonym for publication if desired. Replies by post cannot be given save under exceptional circumstances at the Editor's discretion.**

**2. Letters from Approved Homes in reply to special needs published in the Bureau should state terms and full particulars, and be sent prepaid under cover to the Editor of the Bureau with name written across coupon for identification.**

**3. Proprietors of Homes which have not yet been entered on the List of Approved Homes, but have spare accommodation likely to suit special needs, are invited to write for an application form for registration The fee for registration, which includes two announcements of the Home in the Bureau and other privileges, is 10s.**

**4. All communications to be addressed to the Editor of THE HOSPITAL, 28 Southampton Street, Strand, London, W.C. 2, and marked "Bureau of Information."**

### Starting a Venereal Block.

### Answer to September Question.

By M. B. M.

THE question was :

**It is proposed to start a venereal block, containing two wards of twelve beds each (male and female), a clinic, and an out-patients' department. (a) What nursing staff will be required, and how should it be distributed? (b) What equipment will be necessary for each department?**

### The Nursing Staff.

The number of nurses required to run two wards of twelve beds each would depend really on the plan of the building. If the two wards were on different floors it would require more nurses on day duty to work them efficiently. There is not a great amount of actual nursing to be done as a rule with these particular cases, but it would not do to leave the wards without a nurse within call.

The most efficient arrangement would be for the clinic (assuming this to be the teaching centre) and out-patient department to be arranged so that they were run by the ward staff. In all hospitals this would not be possible, as the departments might become too big; this again would depend on the locality and the radius from which patients would be received. In any case, there should only be one sister in charge of the whole department.

Patients suffering from syphilis and gonorrhœa who apply for treatment will possibly have to go through all three departments—in any case, all patients who become in-patients must attend the out-patient department afterwards to finish their treatment. They will be much more ready to attend and complete their cure if they realise they will only come in contact with the same sister and, possibly, nurse. With patients suffering from this particular disease the object is to complete the cure, and anything that can be arranged to further this end is not only an advantage to the patient, but also to the nation. If the sister gets to know the patient through all stages, she will probably be able to use her influence to this end.

#### THE WARDS.

Assuming, therefore, that the same staff will be in charge of the whole department and that the wards are on the same level, the following nurses would be required :

1. A sister on day duty in complete charge of the whole block. Owing to the peculiar nature of the disease, it would be advisable to have only fully-trained nurses working under the sister. The department would be a distinct branch in itself, and it might lead to difficulties with parents if probationers were put to work in this block. Then, again, it requires experience to note any ill-effects or unforeseen symptoms which may occur after an injection. The nurses must also realise the risk they are running unless they are careful and conscientious. They must be possessed of a large amount of common sense and tact if they are to do their best for the patients.

2. DAY NURSES.—In order that the nurses should get their regular "off-duty" time and leave the wards efficiently staffed, three nurses would be needed on day duty. When the days and hours had been arranged for the various departments to be opened, then the half-days and "off-duty" time for the nursing staff could be fitted in.

Of course, if the hospital was situated in a thickly-populated district and took in patients from a large radius it might not be possible to run the block with such a small staff; this, however, would be exceptional, because the number of beds allotted would regulate to a certain extent the patients treated in the out-patient department—at any rate as far as syphilis is concerned.

3. NIGHT NURSES.—On night duty it would also be necessary to have three nurses. This is generous for night duty, as beyond seeing to the patients' charts and meals there is very little nursing to do, unless any of the patients have a marked "reaction" after the injection of salvarsan. But a nurse would be needed for relieving for "nights off" and meals, so although the staff at some times during the night is more than is actually required, it would not be advisable to do with less.

The spare time could be filled in in many ways, as there are records to be kept, needles and syringes to overhaul, dressings to be prepared, sterilising tins to be filled with towels and swabs, etc., and all this could be done by the night staff, and so save the day nurses, who have their time fully occupied otherwise. It might be possible to do without this third nurse on night duty and obtain the "relief" from another part of the hospital. In this particular work, however, it is not satisfactory to have an "odd" nurse coming in.

4. It might be necessary at times to employ a male nurse, but the work as a rule would not be sufficient to need one all the time.

#### THE CLINIC.

The clinic would not entail a great amount of work to be done by the nursing staff. New patients would be seen by the physician, Wassermann tests taken, arrangements made for new patients to be admitted (in booking-in the patients it is important to allow for complications, so that the beds do not get blocked), and in some cases it might happen that injections would have to be given to patients who were unable to attend the out-patient department.

A sister and a nurse would be quite able to manage this, while the two other nurses would be on duty in the wards.

#### THE OUT-PATIENT DEPARTMENT.

The out-patient department would be for the patients who had already been in-patients or who were being treated for gonorrhœa. On an average 266 patients who had been in-patients would be seen each week if the beds had been kept full. The number of patients with gonorrhœa would vary, however, and would not have been in-patients. In this department the sister and nurse would pre-

(*Continued on column 3, page 2.*)

### QUESTION for OCTOBER.

### AN ISOLATION HOSPITAL DIFFICULTY.

In an isolation hospital wards are often closed for months at a time. In spite of good ventilation and dry weather, the enamel of the bedsteads peels off, and the stoves get rusty very quickly. Explain fully what can be done to prevent this.

### RULES.

The following rules must be observed:—

1. Contributions must be written on one side of the paper. Brevity and terseness are desirable features. The MSS. must bear the name and address of the sender and be accompanied by coupon to be cut from the back cover (inside page) of the current issue of THE HOSPITAL. A pseudonym must be chosen if the name is not to be published.

2. Contributions must be addressed to the Editor of THE HOSPITAL, Institutional Worker Supplement, 28 & 29 Southampton Street, Strand, London, W.C. 2, should reach him before the end of the current month, and be marked in the left-hand corner "Facts and Figures."

**A minimum payment of Half-a-Guinea will be made for each published answer.**

### QUESTIONS INVITED.

IN connection with our Question Box, we offer every month five shillings each for the two best questions which are sent in for consideration. The questions may be on any institutional subject and concern any department, from the secretary's to the porter's, or the matron's to the domestic staff; the one essential is that they must be practical and deal with points that have a definite relation to hospital or institutional administration, upkeep, finance, or management. Points relating to artificers' work, housekeeping, the laundry, out-patients, and other practical matters will be welcome. It is hoped that thus, when difficult or doubtful points arise in the course of their work, institutional workers will be encouraged to put them in the form of a question and send them to the Editor, THE HOSPITAL Bureau of Information, 28 Southampton Street, Strand, W.C. 2, marked "Question Box."

The best questions will be published in due course and our readers will be given the opportunity of answering them. Thus all institutional workers may be able to co-operate with the view of helping the work and smoothing the difficulties of each other. In short, we want the Question Box of the *Institutional Worker* to be freely used by every worker habitually.

### ENQUIRIES AND ANSWERS.

### *EMPLOYMENT AND TRAINING.*

### Free Training in Chiropody.

YOUR best plan is to try to obtain training with a chiropodist who has a private practice. If you write to Mr. Runting, of 7 New Burlington Street, London, W., he will undoubtedly be able to help you. Perhaps it might be possible to obtain training, board, and lodging in return for services given.—R. W.

### Private Hospitals and Nursing Homes in Liverpool.

WE are afraid that your only means of obtaining such a position as you desire is to advertise your needs in the nursing journals which are published weekly—such as the *Nursing Mirror*. We cannot give postal replies. A coupon should be enclosed with each inquiry.—NURSE L. R. R.

### *WAR MATTERS.*

### Post as Motor Driver in France.

YOU might possibly obtain the work that you desire if you apply to the following committees: Wounded Allies Relief Committee, 8 Grosvenor Gardens, S.W. 1; the Women's Legion, 72 Upper Berkeley Street, W. 1; and the Women's Reserve Ambulance, 199 Piccadilly, W. 1.—ANXIOUS TO HELP.

### Help for Soldier's Wife.

THE Sailors' and Soldiers' Families Aid Association, 23 Queen Anne's Gate, S.W. 1, might help the soldier's wife you refer to; at any rate there is no reason why she should not write to them and explain her difficulties and requirement.—WAR WORKER.

### *MISCELLANEOUS.*

### The Value of Waste Dressings.

THE necessity of making use of all waste material is having the effect of stimulating genius. An enterprising manufacturer has discovered the means of utilising waste dressings in national interests. Septic dressings are invariably destroyed in the hospital furnaces. Mr. John D. Marshall, of 50 Wigmore Street, London, W., is circularising the hospitals with a view to making arrangements for the salvage of all dressings. He offers to provide suitable containers with closely-fitting lids, to collect daily, leaving clean containers for further use, and to pay at the rate of £2 per ton. He states that this will be possible only if the large hospitals allow him to purchase their waste dressings, so that the quantity collected may be sufficient to usefully employ the requisite machinery. We do not know in what direction it is intended to use these dressings after treatment, but Mr. Marshall undertakes the responsibility of properly dealing with them. The question seems to merit the attention of hospital secretaries.—H. W.

### *THE HOUSEKEEPERS' DEPARTMENT.*

### Meat Supply and Cold Storage.

THERE is no doubt that relatively small institutions in London can effect a saving in the cost of meat by purchasing, at any rate, the greater portion of it in the market. In these days when so many are only too anxious to serve the community by helping the hospitals, some friend can be found having expert knowledge in buying meat who will gladly give his services in this direction. Experience seems to show that it is necessary, when purchasing meat in this way, to estimate at the end of the week what will be required during the ensuing week, and to place orders with the agent in advance for the delivery of meat on certain days accordingly. In the absence of a cold store this method necessarily creates a difficulty, for inasmuch as numbers fluctuate it is possible to over-estimate the amount required, and further, when purchasing carcasses it is not easy to keep within the limit of weight desired. The provision of a cold store overcomes this difficulty, for any surplus can be kept until required. The best type is that reduced in temperature by mechanical means. The cost, though higher now than in pre-war days, provided a small room is available for the purpose, is not nearly so great as it is generally supposed to be; a surprisingly large quantity of meat can be packed into a small space. The smaller the store the less will be the cost of machinery and maintenance. Cold storage is also required for milk, butter, and the like, and it is possible to have an airtight cubicle in the main cold store for this purpose. Ice required for medical purposes can be made in the course of reducing the temperature of the store. The desirability, therefore, of installing a cold store is indicated from an economical standpoint. Messrs. Ernest West and Beynon, Ltd., 82 Belvedere Road, Lambeth, S.E., specialise in cold stores which are eminently suitable for hospital purposes.—STEWARD.

### STARTING A VENEREAL BLOCK.

(*Continued from page 1.*)

pare, chaperon, and in some cases give injections and douches to the women patients.

It would be advisable to change the nurses every six months, unless they preferred to stay longer. They would then have three months on day duty and three months on night duty—*i.e.*, a new nurse would be required every alternate month. Of course, in a small hospital this would be impossible to arrange—in that case an extra nurse for part of the year would be needed to supply the holidays. In addition to the nursing staff a woman would be required as wardmaid.

(*To be continued.*)

